# Association of probiotic ingestion with serum sex steroid hormones among pre- and postmenopausal women from the NHANES, 2013–2016

**DOI:** 10.1371/journal.pone.0294436

**Published:** 2023-11-16

**Authors:** Siying Zou, Xu Yang, Nihong Li, Hong Wang, Junhao Gui, Junjun Li

**Affiliations:** Reproductive Medicine Center, ChengDu Fifth People’s Hospital, ChengDu, Sichuan, China; UFRN: Universidade Federal do Rio Grande do Norte, BRAZIL

## Abstract

**Background:**

Sex hormone-related diseases, encompassing a wide range of conditions from reproductive disorders to certain cancers, pose significant health challenges worldwide. Recent scientific investigations have highlighted the intricate interplay between the gut microbiome and sex hormone regulation, indicating the potential for microbiota-targeted interventions in the management of such diseases. Although individual studies have elucidated the influence of the gut microbiome on sex hormones, a comprehensive cross-sectional examination of the population-wide prevalence of probiotic intake and its correlation with sex hormones is still lacking.

**Objectives:**

This study aimed to evaluate the association of probiotic ingestion with sex hormones in pre- and post-menopausal women.

**Methods:**

We conducted an observational cohort study comprising a nationally representative sample of adults who participated in the National Health and Nutrition Examination Survey between 2013 and 2016. Probiotic ingestion was considered when a subject reported yogurt or probiotic supplement consumption during the 24-h dietary recall or during the Dietary Supplement Use 30-Day questionnaire. A survey-weighted generalized linear model was used to analyze the association between probiotic intake and female/male sex hormones. To reduce selection bias, we used propensity score matching (PSM).

**Results:**

This study included 2,699 women, with 537 of them consuming yogurt and/or dietary supplements containing probiotics, while the remaining 2,162 women did not consume any probiotics. The findings indicated that there were associations between probiotic intake and sex hormone levels in premenopausal and postmenopausal women. For premenopausal women, probiotic intake was positively associated with estradiol (E_2_) levels. On the contrary, in postmenopausal women, probiotic intake was inversely associated with total testosterone (TT) levels.

**Conclusions:**

This study indicated that probiotic consumption was associated with higher E2 level in premenopausal women and lower TT level in postmenopausal women. Probiotic intake might be a sensible strategy for preventing sex hormone-related diseases.

## Introduction

Sex hormones play a pivotal role in both the initiation and maintenance of female reproductive health [1, 2]. Total testosterone (TT), estradiol (E_2_), and sex hormone-binding globulin (SHBG, a protein that binds to sex hormones) have been shown to regulate crucial physiological processes within the human body [1, 2]. Although testosterone serves as a precursor for estradiol biosynthesis and exerts androgenic effects, the influence of estrogen extends to diverse biological systems, encompassing the nervous, vascular, and skeletal domains [3–5]. Moreover, sex hormone-binding globulin, with its role in transporting androgens and estrogens, intricately contributes to the cyclical nature of male and female sex hormone dynamics, serving as a regulator for endocrine balance [6].

Disruptions in sex hormone homeostasis can lead to a spectrum of adverse health outcomes, encompassing conditions such as infertility and polycystic ovary syndrome (PCOS) among premenopausal women, as well as osteoporosis, cardiovascular disorders, and menopausal symptoms among postmenopausal women [7–10]. Beyond these immediate effects, mounting evidence underscores the broader impact of sex hormone-related disorders on a range of diseases, including neuropsychiatric disorders and various types of cancers [11, 12]. Consequently, these disorders bear the potential to exacerbate disease burdens in the female population.

The human gut, a reservoir teeming with trillions of microorganisms collectively referred to as the gut microbiota, constitutes a dynamic ecosystem with symbiotic ties to its human host [13]. This intricate microbiota has evolved in tandem with its human counterpart, contributing indispensable roles in safeguarding, metabolizing, and structurally supporting the host’s well-being [14]. A previous review has suggested the potential roles of gut microbiota in sex hormone-related diseases that affect women, including ovarian cancer, postmenopausal osteoporosis, PCOS, and type 1 diabetes [15].

Mechanisms elucidating the impact of the gut microbiota on sex hormone-related diseases involve complex interactions and modulation. Recent studies have delved into these mechanisms, highlighting the capacity of probiotics to exert influence on the composition and function of the gut microbiota [16, 17]. These interactions, encompassing intricate microbial-host crosstalk, metabolite production, and modulation of inflammatory pathways, contribute to the potential of probiotics to alleviate associated diseases [18, 19]. Factors such as microbial diversity, metabolite profiles, and the balance between beneficial and pathogenic microorganisms all intertwine in shaping the intricate network of the effects mediated by probiotics [20–24]. Probiotics, recognized for their ability to modulate the gut microbiota, offer a promising avenue for addressing associated diseases. However, despite the mounting interest, the evidence linking probiotic intake to sex hormones in premenopausal and postmenopausal women remains limited.

Hence, this study aims to unravel the intricate relationship between probiotic consumption, whether through yogurt or supplements, and sex hormones. By leveraging a robust dataset from the National Health and Nutrition Examination Survey (NHANES) for the years 2013–2016, the study aims to evaluate the potential of probiotic interventions in mitigating and preventing sex hormone-related diseases. As a pivotal step in understanding the role of probiotics in female health, these findings could pave the way for novel strategies to ameliorate the burdens associated with sex hormone-related disorders.

## Materials and methods

### Study design and settings

We designed a cross-sectional analysis using data from the NHANES [25]. The National Health and Nutrition Examination Survey (NHANES) is a nationally representative dataset conducted by the Centers for Disease Control and Prevention (CDC) in the United States. NHANES is designed to assess the health and nutritional status of the U.S. population through comprehensive surveys and physical examinations. NHANES employs a complex, multistage sampling design to ensure that its sample is representative of the non-institutionalized civilian population of the United States. This sampling methodology involves selecting participants from various demographic and geographic strata to ensure a diverse and accurate representation of the population. Notably, NHANES includes a relatively large sample size, typically involving several thousand participants, and collects data on a wide range of demographic factors, including age, gender, race, ethnicity, income, education, and geographic region. NHANES data are publicly available, promoting transparency and facilitating scientific research across various fields. Due to its careful sampling design, NHANES is considered representative of the non-institutionalized civilian population in the United States. This indicates that findings derived from NHANES data can be generalized to the broader U.S. population within certain statistical margins.

### Study population

**Fig 1:**
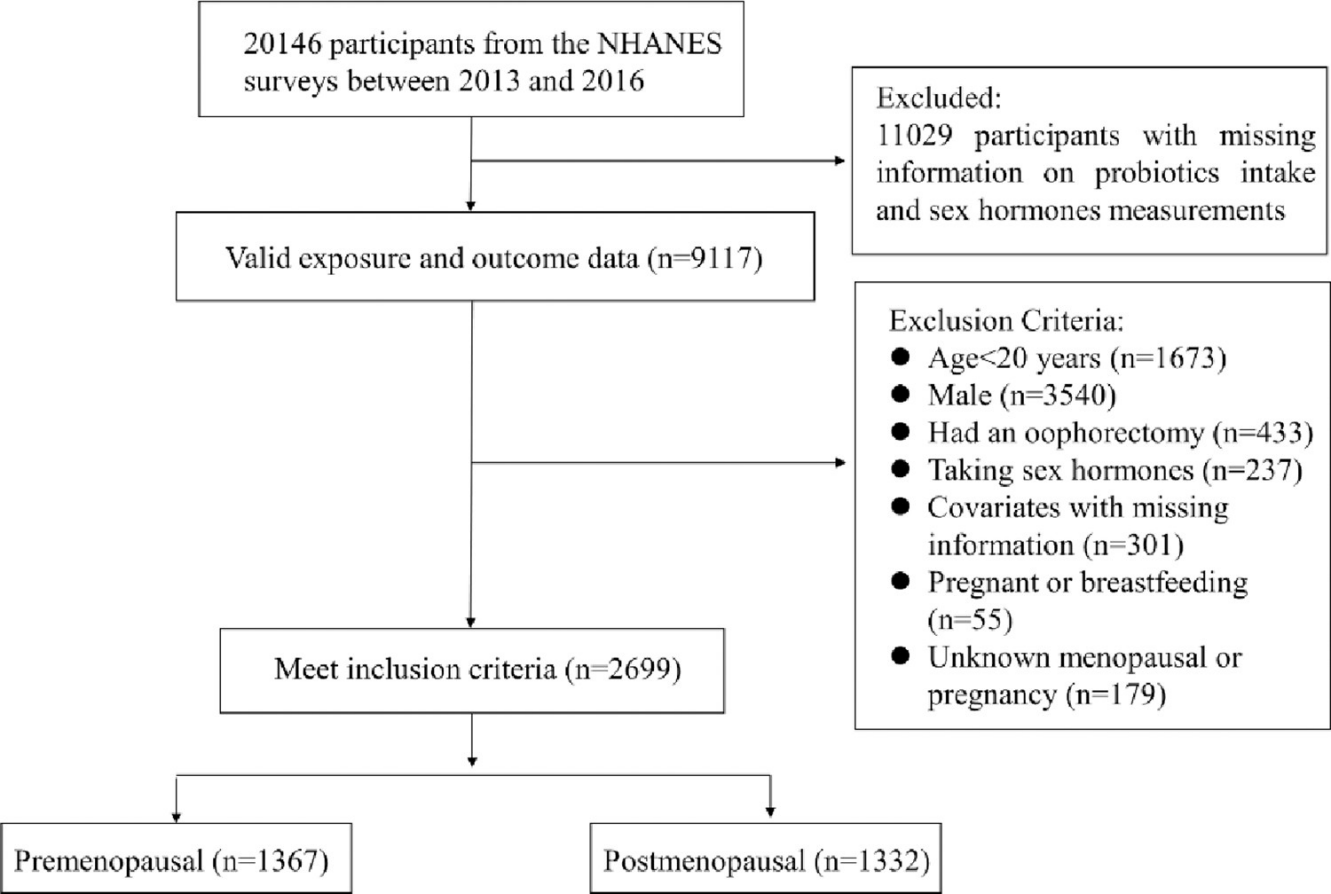
Flowchart of participant selection.

The present study collected data from two cycles (2013–2014 and 2015–2016) from the NHANES. Individuals (n = 9,117) with available data on sex hormones and probiotic intake were initially included. Individuals aged < 20 years at interviews (n = 1,673); men (n = 3,540); and women who had undergone oophorectomy (n = 433), or were taking sex hormone-containing medications (hormonal contraceptives, hormone therapy, etc.; n = 237) were excluded from the study. Additionally, women with missing information on covariates (n = 301) were excluded. Menopausal status was determined based on the responses to the questionnaire on reproductive health, asking the question “Have you had at least one menstrual period in the past 12 months?” (Please do not include bleedings caused by medical conditions, hormone therapy, or surgeries)” [26]. Women who answered “No” were considered postmenopausal and those who answered “Yes” were considered premenopausal. Subjects who answered “No” to the first question were subsequently asked, “What is the reason that you have not had a period in the past 12 months? (Options: pregnancy; breastfeeding; hysterectomy; menopause/change of life; other)” [26]. Consequently, women who had not had a period in 12 months due to pregnancy or breastfeeding (n = 55), or women who had an unknown pregnancy or menopausal status were excluded (n = 179). If women are initially classified as premenopausal based on a questionnaire survey but are over 55 years old, they will be reclassified as postmenopausal [27]. On the other hand, for women under the age of 40 who are initially classified as postmenopausal according to a questionnaire survey, they will only remain classified as postmenopausal if their estrogen levels are less than 40.8 pg/ml; otherwise, they will be reclassified as premenopausal women [28]. The final analytic sample contained 1367 premenopausal women and 1332 postmenopausal women. Fig 1 presents a flowchart of participant selection.

### Assessments of probiotic intake

This study considered yogurt or probiotic-containing supplements as probiotic intake [29–31]. We used the First Day Dietary Interview and Second Day Dietary Interview to assess yogurt intake. The first dietary interview was conducted in person at the Mobile Examination Center, and the second interview was conducted via telephone 3–10 days later. The mean of the nutritional information from both the in-person recall and the telephone recall was utilized. To assess probiotic supplements intake, we used the 30-Day Dietary Supplement Use questionnaire, which collected personal interview information on prescription and non-prescription dietary supplements 30 days before the interview date.

### Assessments of serum sex hormones

Isotope dilution high-performance liquid chromatography–tandem mass spectrometry was used to measure serum TT and E_2_ levels [32]. The chemiluminescence method was used to analyze the reaction products of SHBG and immune antibodies to determine the concentration of SHBG [33]. The testosterone-to-estradiol ratio (TT/E2) was determined by dividing the picomolar concentration of testosterone by that of estradiol. By using the formulas below created by Vermeulen et al. and described by Ho et al., the free testosterone (FT) and free androgen index (FAI) were calculated [34, 35].


$$T_{free}=T_{total}-N-S+\frac{\sqrt{\begin{array}{c} {\left(N+S-T_{total}\right)}^{2}+4{NT}_{\_total} \end{array}}}{2N}$$

$$FAI=T_{total}*\frac{100}{S}$$

N=0.5217*Albumin+1

S=SHBG

### Covariates

Based on a previous study [26, 27], we selected a set of covariates, including age, race/ethnicity, education level, body mass index (BMI), poverty–income ratio (PIR), smoking status, and alcohol consumption status. Six-month period variables and the time of blood collection were also added to account for seasonal and time differences in sex hormone levels. Age was classified into three levels: 20–39, 40–59, and ≥60 years. Race was classified into two levels: Hispanic and non-Hispanic. BMI was classified into three levels: normal weight (<25 kg/m^2^), overweight (25–30 kg/m^2^), and obese (≥30 kg/m^2^). PIR was categorized into four quantiles: Q1 (0–1.080), Q2 (1.080–2.080), Q3 (2.080–3.955), and Q4 (≥3.955) [36]. Smoking status was assessed based on the serum cotinine level, which was categorized into two groups: 1) a serum cotinine level less than the limit of detection (LOD) (0.015 ng/mL) and 2) equal to or greater than the LOD [2]. Alcohol consumption status was defined as none, moderate (1 drink/day for women), and heavy (2–3 drinks/day for women) based on definitions from the National Institute on Alcohol Abuse and Alcoholism in the National Institute of Health.

### Statistical analysis

Owing to the complex design of the NHANES survey, an appropriate sampling weight (1/2*WTDR2D for 2013–2016) was used while analyzing the data to produce representative and unbiased statistics. These weights were applied to each participant’s data during analysis to ensure that the results are representative of the general population. The weights account for variations in selection probabilities and nonresponse rates within the survey design. The baseline characteristics for categorical variables are presented as percentages, whereas those of continuous variables are presented as means and standard deviations. We compared baseline characteristics stratified based on probiotic intake status using the weighted chi-square test for categorical variables and the weighted linear regression model for continuous variables.

Survey-weighted generalized linear model stratified based on menopausal status was used to assess the β coefficient of the association between probiotic intake (exposure) and sex hormones (outcome). A survey-weighted generalized linear model was adjusted for potential confounders. No factors were adjusted in Model 1, and all covariates were adjusted in Model 2. Moreover, propensity score matching (PSM) was used to avoid potential bias arising from differences in baseline characteristics [37]. This study selected confounding factors, such as age, race, education level, six-month period variable, and the time of blood collection, for matching. The PSM was conducted at a 1:1 ratio with a caliper value of 0.02. After PSM, we re-analyzed the association between probiotic exposure and sex hormones.

We used SPSS version 26.0 for PSM and EmpowerStats for statistical analysis. A two-tailed p-value of <0.05 was considered statistically significant.

## Results

### Baseline characteristics of the study cohort according to probiotic intake

This study included 2,699 women aged ≥20 years. Table 1 describes their baseline characteristics according to menopausal status and probiotic intake. In this study cohort, 537 women consumed yogurt and/or probiotic supplements, among them, 454 women only had yogurt, 59 women only had probiotic supplements, and 24 women had both, while the remaining 2,162 women consumed none. Women with probiotic exposure were more likely to be 40–59-years-old, non-Hispanic, and non-smokers; have low BMI; and have higher income and educational levels.

**Table 1:** Characteristics of participants in the NHANES (2013–2016) according to menopausal status and probiotic intake.

	Premenopausal (N = 1367)			Postmenopausal (N = 1332)			Total		
Demographic characteristics	No Exposure to Probiotics	Exposure to Probiotics	*p* value	No Exposure to Probiotics	Exposure to Probiotics	*p* value	No Exposure to Probiotics	Exposure to Probiotics	*p* value
	N = 1098	N = 269		N = 1064	N = 268		N = 2162	N = 537	
Average age	34.35 ± 8.80	36.72 ± 8.77	<0.001*	61.10 ± 11.11	61.26 ± 10.68	0.808	47.11 ± 16.67	49.38 ± 15.70	0.002*
Age(%)			0.003*			0.522			0.003*
20–39	69.87	60.95		1.97	1.24		37.48	30.15	
40–59	30.13	39.05		46.92	45.09		38.14	42.17	
≥60	~	~		51.11	53.67		24.38	27.69	
Race			0.023*			0.118			0.003*
Hispanic	23.54	17.52		12.05	8.98		18.06	13.12	
Non-Hispanic	76.46	82.48		87.95	91.02		81.94	86.89	
Education Level			<0.001*			<0.001*			<0.001*
Less than high school and high school	37.28	13.12		44.153	25.291		40.56	19.40	
Some college or AA degree	35.70	40.49		34.43	35.34		35.09	37.83	
College graduate or above	27.02	46.39		21.41	39.37		24.35	42.77	
Ratio of family income to poverty			<0.001*			<0.001*			<0.001*
Q1	26.65	12.91		17.65	11.28		22.36	12.07	
Q2	25.87	19.10		23.30	18.62		24.64	18.85	
Q3	26.29	24.48		27.50	27.75		26.87	26.17	
Q4	21.19	43.51		31.55	42.35		26.14	42.91	
BMI			<0.001*			0.093			<0.001*
<25 kg/m2	32.67	37.02		24.14	29.64		28.60	33.21	
≥25kg/m^2^-<30kg/m^2^	23.49	32.98		28.90	28.84		26.07	30.85	
≥30 kg/m^2^	43.84	29.99		46.96	41.53		45.33	35.94	
Drinking status			0.111			0.373			0.044*
None	18.97	14.00		18.65	14.90		18.82	14.46	
moderate	22.30	24.39		31.43	34.49		26.66	29.60	
heavy	51.37	56.01		34.21	35.74		43.18	45.56	
NA	7.36	5.60		15.71	14.87		11.34	10.38	
Cotinine			<0.001*			<0.001*			<0.001*
≥LOD	68.60	53.65		61.81	40.31		65.36	46.77	
<LOD	31.40	46.35		38.19	59.69		34.64	53.24	
Six-month time period			0.004*			0.220			0.003*
November 1 through April 30	48.40	39.22		43.83	40.06		46.22	39.65	
May 1 through October 31	51.60	60.78		56.17	59.94		53.78	60.35	
Time of blood draw			0.013*			0.361			0.087
Morning	44.25	35.28		46.49	47.76		45.32	41.72	
Afternoon	33.05	36.39		39.93	36.26		36.34	36.33	
Evening	22.70	28.33		13.58	15.98		18.35	21.96	
Sex hormones									
TT (ng/DL)	27.05 ± 20.13	30.49 ± 53.28	0.084	21.19 ± 13.34	19.51 ± 10.91	0.034*	24.25 ± 17.47	24.83 ± 38.29	0.597
E2 (pg/mL)	97.00 ± 88.45	131.42 ± 151.59	<0.001*	13.93 ± 29.80	15.76 ± 33.42	0.339	57.37 ± 78.97	71.75 ± 122.64	<0.001*
SHBG (nmol/L)	69.35 ± 45.23	80.26 ± 48.48	<0.001*	68.18 ± 38.86	66.85 ± 33.43	0.568	68.79 ± 42.32	73.34 ± 41.94	0.016*
FT (nmol/ml)	11.82 ± 9.24	11.57 ± 20.05	0.748	8.95 ± 5.75	8.56 ± 5.76	0.284	10.45 ± 7.90	10.02 ± 14.63	0.329
FAI	1.83 ± 1.59	1.65 ± 2.35	0.124	1.33 ± 0.94	1.32 ± 1.10	0.872	1.590 ± 1.342	1.480 ± 1.821	0.094
TT/E2	5.65 ± 12.50	6.27 ± 20.44	0.515	37.15 ± 32.42	36.58 ± 31.82	0.776	20.68 ± 28.82	21.90 ± 30.89	0.348

* statistically significant.

### Characteristics of participants after matching based on probiotic intake

The prevalence of most covariates was significantly different (*p* < 0.05) between those with and those without probiotic intake in pre- and post- menopausal women. Based on PSM, The covariates selected for matching such as age, race, education level, six-month period variable, and the time of blood collection of participants between the two groups did not differ significantly (Table 2).

**Table 2:** Characteristics of participants in the NHANES (2013–2016) according to menopausal status and probiotic intake after PSM.

	Premenopausal			Postmenopausal		
Demographic characteristics	No Exposure to Probiotics	Exposure to Probiotics	*p* value	No Exposure to Probiotics	Exposure to Probiotics	*p* value
Average age	35.63 ± 9.03	36.72 ± 8.77	0.044*	61.92 ± 11.30	61.262 ± 10.677	0.329
Age(%)			0.494			0.945
20–39	62.98	60.95		1.47	1.24	
40–59	37.02	39.05		45.23	45.09	
≥60	~	~		53.30	53.67	
Race			0.828			0.533
Hispanic	17.02	17.52		10.11	8.98	
Non-Hispanic	82.98	82.48		89.89	91.02	
Education Level			0.665			0.568
Less than high school and high school	15.01	13.12		27.88	25.29	
Some college or AA degree	39.15	40.49		35.35	35.34	
College graduate or above	45.85	46.39		36.77	39.37	
Ratio of family income to poverty			0.147			0.610
Q1	12.64	12.91		10.07	11.28	
Q2	20.43	19.10		21.67	18.62	
Q3	29.60	24.48		27.84	27.75	
Q4	37.33	43.51		40.42	42.35	
BMI			<0.001*			0.538
<25 kg/m2	41.12	37.02		30.86	29.64	
≥25kg/m^2^-<30kg/m^2^	19.87	32.98		30.94	28.84	
≥30 kg/m^2^	39.02	29.99		38.20	41.53	
Drinking status			0.037*			0.731
None	17.17	14.00		16.37	14.90	
moderate	28.30	24.39		34.49	34.49	
heavy	47.23	56.01		36.45	35.74	
NA	7.30	5.60		12.69	14.87	
Cotinine			0.369			0.185
≥LOD	56.38	53.65		44.35	40.31	
<LOD	43.62	46.35		55.65	59.69	
Six-month time period			0.920			0.164
November 1 through April 30	39.52	39.22		35.89	40.06	
May 1 through October 31	60.48	60.78		64.11	59.94	
Time of blood draw			0.626			0.055
Morning	38.13	35.28		45.19	47.76	
Afternoon	34.76	36.39		42.67	36.26	
Evening	27.11	28.33		12.15	15.98	
Sex hormones						
TT (ng/DL)	26.68 ± 23.68	30.493 ± 53.284	0.141	21.41 ± 14.04	19.51 ± 10.91	0.013*
E2 (pg/mL)	97.17 ± 79.87	131.42 ± 151.59	<0.001*	13.68 ± 32.79	15.76 ± 33.42	0.311
SHBG (nmol/L)	76.19 ± 50.80	80.26 ± 48.48	0.181	70.08 ± 39.90	66.85 ± 33.43	0.150
FT (nmol/ml)	11.19 ± 10.39	11.57 ± 20.05	0.707	8.89 ± 5.92	8.56 ± 5.76	0.362
FAI	1.713 ± 1.69	1.65 ± 2.35	0.629	1.31 ± 0.95	1.32 ± 1.10	0.856
TT/E2	6.19 ± 16.26	6.27 ± 20.44	0.946	37.22 ± 27.61	36.58 ± 31.82	0.729

* statistically significant.

### Correlation analysis of probiotic intake and serum sex hormones before and after matching

Table 3 presents the associations between probiotic intake and sex hormones. For the unadjusted model, among premenopausal women, E_2_ and SHBG levels were higher in participants with probiotic intake than in those without probiotic intake (β = 34.42, 95% confidence interval [CI] 21.08 to 47.76; *p* < 0.001 vs. β = 10.91, 95% CI: 5.15 to 16.66; *p* < 0.001). In postmenopausal women, participants with probiotic intake had lower TT levels than those without probiotic intake (β = −1.68, 95% CI: −3.23 to −0.13; *p* = 0.033). After adjusting for potential confounders, among premenopausal women, E_2_ levels in the probiotic exposed group remained higher than nonexposed group (β = 26.51, 95% CI: 12.61 to 40.42; *p* < 0.001). Among postmenopausal women, the TT in the probiotic exposed group no longer lower than nonexposed group (β = −1.46, 95% CI: −3.03 to 0.12; *p* = 0.071). The SHBG level in the probiotic exposed group was lower than nonexposed group (β = −4.50, 95% CI: −8.73 to −0.25; *p* = 0.038). For the adjusted and unadjusted model, FT, FAI and TT/E_2_ had no statistical difference between those with and those without probiotic intake in pre- and post- menopausal women.

**Table 3:** Association between probiotic intake and sex hormones before matching.

	Model 1	95% CI	*p* value	Model 2	95% CI	*p* value
prememopausal	β			β		
TT	3.45	−0.46 to 7.36	0.084	5.33	1.27 to 9.39	0.010
E2	34.42	21.08 to 47.76	<0.001*	26.51	12.61 to 40.42	<0.001*
SHBG	10.91	5.15 to 16.66	<0.001*	3.81	−1.78 to 9.40	0.182
FT	−0.26	−1.84 to 1.32	0.748	1.36	−0.24 to 2.96	0.097
FAI	−0.18	−0.40 to 0.05	0.124	0.11	−0.12 to 0.33	0.348
TT/E2	0.61	−1.23 to 2.46	0.515	1.30	−0.63 to 3.24	0.187
postmenopausal						
TT	−1.68	−3.23 to −0.13	0.033*	−1.46	−3.03 to 0.12	0.071
E2	1.83	−1.92 to 5.58	0.339	1.77	−1.95 to 5.49	0.351
SHBG	−1.33	−5.90 to 3.23	0.568	−4.50	−8.73 to −0.25	0.038*
FT	−0.38	−1.08 to 0.32	0.284	0.02	−0.67 to 0.70	0.966
FAI	−0.01	−0.13 to 0.11	0.872	0.06	−0.05 to 0.18	0.274
TT/E2	−0.57	−4.50 to 3.35	0.776	−0.58	−4.30 to 3.14	0.759

Model 1: No adjustments made for confounding factors.

Model 2: Adjustments made for all the confounding factors.

* statistically significant.

Table 4 shows the associations between probiotic intake and sex hormones after matching. In the unadjusted analysis, a higher E_2_ level was observed in the probiotic-exposed group among premenopausal women (β = 34.25, 95% CI: 19.40 to 49.09; *p* = 0.012), and a lower TT level was observed in the probiotic-exposed group among postmenopausal women (β = −1.90, 95% CI: −3.40 to −0.41; *p* = 0.013). In the adjusted analysis, among premenopausal women, E_2_ levels remained higher in the probiotic-exposed group after adjusting for potential confounders (β = 28.75, 95% CI: 14.21 to 43.29; *p* < 0.001). Among postmenopausal women, TT levels remained lower in the probiotic-exposed group (β = −2.14, 95% CI: −3.61 to −0.67; *p* = 0.004). And after matching, for the adjusted and unadjusted model, the FT, FAI and TT/E_2_ had no statistical difference between those with and those without probiotic intake in pre- and post- menopausal women.

**Table 4:** Association between probiotic intake and sex hormones after matching.

	Model 1	95%CI	*p* value	Model 2	95%CI	*p* value
prememopausal	β			β		
TT	3.81	−1.25 to 8.88	0.141	1.70	−3.21 to 6.62	0.498
E2	34.25	19.40 to 49.09	0.012*	28.75	14.21 to 43.29	<0.001*
SHGB	4.06	−1.88 to 10.01	0.181	0.99	−4.37 to 6.36	0.717
FT	0.38	−1.58 to 2.33	0.707	0.06	−1.82 to 1.93	0.952
FAI	−0.06	−0.30 to 0.19	0.629	−0.04	−0.27 to 0.19	0.710
TT/E2	0.08	−2.15 to 2.31	0.946	−0.28	−2.48 to 1.93	0.806
postmenopausal						
TT	−1.90	−3.40 to −0.41	0.013*	−2.14	−3.61 to −0.67	0.004*
E2	2.08	−1.94 to 6.09	0.311	1.95	−1.88 to 5.78	0.319
SHGB	−3.23	−7.63 to 1.17	0.150	−3.29	−7.21 to 0.63	0.100
FT	−0.33	−1.03 to 0.38	0.362	−0.41	−1.23 to −1.07	0.218
FAI	0.01	−0.11 to 0.14	0.856	−0.00	−0.12 to 0.11	0.953
TT/E2	−0.65	−4.29 to 3.00	0.729	−0.08	−0.04 to −3.43	0.963

Model 1: No adjustments made for confounding factors.

Model 2: Adjustments made for all the confounding factors.

* statistically significant.

## Discussion

To the best of our knowledge, this is the first study to estimate the associations between probiotic intake and sex hormones in the study population of the NHANES 2013–2014 and 2015–2016. The study introduces a novel perspective by examining the correlation between probiotic consumption and sex hormone levels within a population-based sample. This research notably addresses a significant gap by considering both premenopausal and postmenopausal women, thereby shedding light on how probiotics might influence hormone regulation during different physiological stages. The study’s distinctive feature lies in its pioneering exploration of the complex interplay between probiotic intake and sex hormone levels within a nationally representative population. Leveraging data from the NHANES database, we have achieved a high participant count, bolstering the reliability of their conclusions. By investigating a spectrum of sex hormones, including E_2_ and TT, the study contributes to a comprehensive comprehension of the association between probiotics intake and sex hormones levels in women. This large, nationally representative sample of America women revealed that premenopausal women who consumed probiotics had higher E_2_ levels and postmenopausal women who consumed probiotics had lower TT levels than women who did not consume probiotics. Moreover, this result did not change after adjustment for age, demographic characteristics, BMI, and smoking and alcohol consumption statuses.

The study’s findings carry implications for managing hormone-related disorders in women, particularly those undergoing menopause. By revealing associations between probiotic intake and sex hormone levels, this study provides a foundation for exploring probiotics as a potential therapeutic avenue for hormone-related conditions. Menopause and hormonal fluctuations can lead to various symptoms that affect women’s quality of life. The results suggest that probiotic consumption might be a novel approach to alleviating menopausal symptoms, potentially improving women’s overall well-being during this life stage. The study’s discovery of lower testosterone levels in postmenopausal women consuming probiotics is particularly noteworthy due to the association between elevated testosterone levels and cardiovascular risk. With millions of women entering menopause each year, the potential public health impact of these findings is substantial. Exploring novel, safe, and accessible interventions such as probiotics aligns with the broader goal of enhancing women’s health and well-being. The study contributes to building the scientific foundation for using probiotics as a potential tool for managing hormonal imbalances and associated health conditions. This evidence-based approach can guide healthcare professionals and researchers in developing targeted interventions.

However, previous studies about the association between probiotic consumption and sex hormones revealed different results. Bonorden et al. reported that a 2-month intervention with probiotic capsules did not significantly alter plasma hormone concentrations in premenopausal women [38]. These inconsistent findings might be attributable to the variations in study designs, (e.g., cross-sectional studies vs. randomized, placebo-controlled trial), participant characteristics, probiotic types, and dosage. Our analysis revealed that premenopausal women who consumed probiotics had higher E_2_ levels. Moreover, this study was based on a national survey with a large sample size, which could offer reliable evidence. Additionally, the specific linear correlations between gut microbiota and serum hormone levels supported our results [39]. The gut microbiota that can metabolize estrogens is also termed as the estrobolome. The microbiota can produce β-glucuronidase, an enzyme that can deconjugate estrogens [40, 41] and has been shown to increase local and systemic estrogen levels [42, 43]. The gut microbiome is relatively diverse, and its interactions with hormones may be rather complex. It is not the sole determinant of hormone metabolism. Other factors, including diet, genetics, and host physiology, contribute to the overall hormonal balance. Therefore, more mechanistic studies are needed to establish a clear cause-and-effect relationship between specific probiotics and estrogen metabolism. Different probiotic strains might influence the estrobolome differently. Some strains may enhance estrogen metabolism, while others might not have the same impact. This underscores the importance of understanding strain-specific effects. Notably, the strains recognized for modulating gut-brain interactions, such as *Lactobacillus rhamnosus GG* and *Bifidobacterium* has demonstrated the capacity to regulate the hypothalamic-pituitary-adrenal axis [44]. By extrapolating from prior research on these and other pertinent strains, we hypothesize the potential of certain probiotics to indirectly modulate sex hormone levels via gut microbiota-mediated mechanisms. As a result, this mechanism provided theoretical support for the positive correlation between probiotic intake and estrogen.

However, we found no positive association between probiotic intake and E_2_ level in postmenopausal women. This difference might be attributable to the physiological condition of the study population. A previous study revealed that the gut microbiota composition differs between pre- and postmenopausal women [45]; thus, the effect of probiotic intake on sex hormones also differs. Our results are supported by a randomized clinical study conducted by Szydłowska et al., which revealed that probiotic intake does not affect E2 levels in perimenopausal and postmenopausal women [46]. Further, they revealed that follicle-stimulating hormone (FSH) levels significantly increased after probiotic intake. However, our study data were obtained from the NHANES database, which had no data about FSH. Hence, we cannot verify these results, and this is a limitation of our study.

A total of 1.5 million women enter menopause annually. Discovering innovative and safe therapies to improve the quality of life after menopause is a challenge in modern society. However, there is limited research on the effects of probiotics on hormones in postmenopausal women. For the first time, we revealed that postmenopausal women who consumed probiotic supplements and yogurt had lower TT levels. Several meta-analyses of randomized controlled trials supported our results, demonstrating that probiotic supplementation significantly decreases TT levels in women with PCOS [47, 48]. The modulation of TT by probiotics may be linked to several mechanisms, including their capacity to reduce inflammation in the gut and ultimately reduce insulin levels and insulin resistance that stimulates testosterone secretion [49, 50]. This is a particularly useful finding. A study has also revealed that higher levels of testosterone are associated with increased cardiovascular disease and coronary heart disease in postmenopausal women [51]. Thus, our study can provide evidence for the application of probiotics in postmenopausal women.

### Strengths and limitations

Our study had several strengths. The analysis was based on a large cross-sectional survey representative of the US population. To the best of our knowledge, this is the first cross-sectional study to assess the association between probiotic ingestion, through probiotic supplements or yogurt, and female sex hormones. We also developed an analytic strategy wherein PSM was conducted to adjust for confounders such as age, race, educational level, six-month period variable, and the time of blood collection. However, our study has some limitations. First, the cross-sectional study design indicates that we cannot deduce causality. The association between probiotics intake and sex hormones levels in women may be bidirectional. And to address this limitation, future prospective longitudinal studies are needed, which could enable us to monitor changes in probiotic intake and sex hormone levels over time, offering a clearer understanding of the temporal relationship between these variables. Second, the absence of data on gonadotropins (luteinizing hormone and FSH) hindered us from drawing a clearer picture of the possible biological mechanisms. These hormones are pivotal in regulating reproductive processes and sex hormone production. We speculate the impact of altered gonadotropin levels on menstrual cycle regulation, spermatogenesis, and sex hormone balance. Furthermore, potential interplay within the hypothalamic-pituitary-gonadal axis and bidirectional connections between gonadotropins and immune responses could be explored. Future research directions could involve targeted measurement of gonadotropin levels, animal models to elucidate downstream effects, and molecular profiling to understand signaling cascades. Another limitation is the potential for misclassification of menopause status. Variability in the accuracy of self-reported menopause status could impact the categorization of participants. Furthermore, we also acknowledge the concerns raised regarding the timing and measurement of estradiol in pre-menopausal women. The lack of standardized timing for sample collection during the menstrual cycle introduces the potential for misclassification of hormone levels and hinders accurate interpretation of hormonal profiles.

## Conclusions

This study revealed that the ingestion of probiotic supplements or yogurt was associated with higher E2 levels in premenopausal women and lower TT levels in postmenopausal women. Probiotic ingestion might be a sensible strategy for treating sex hormone-related diseases.
